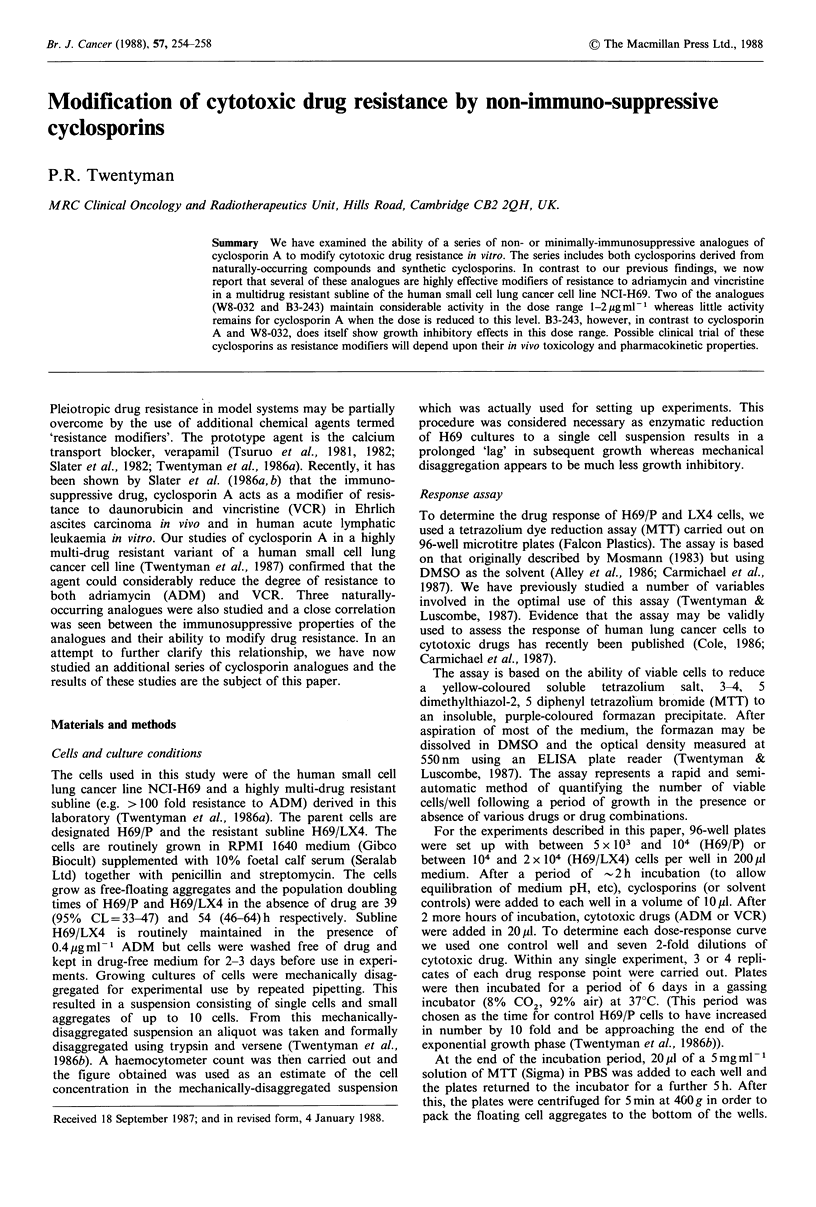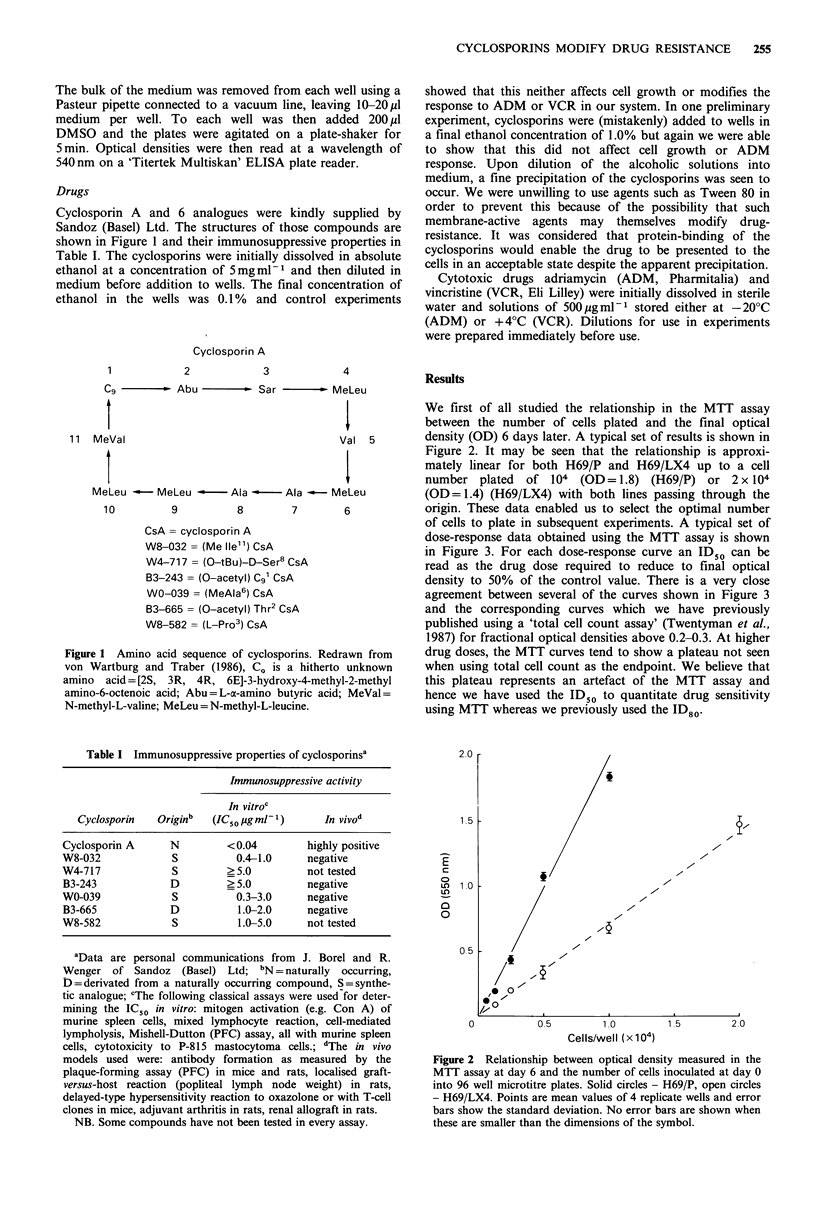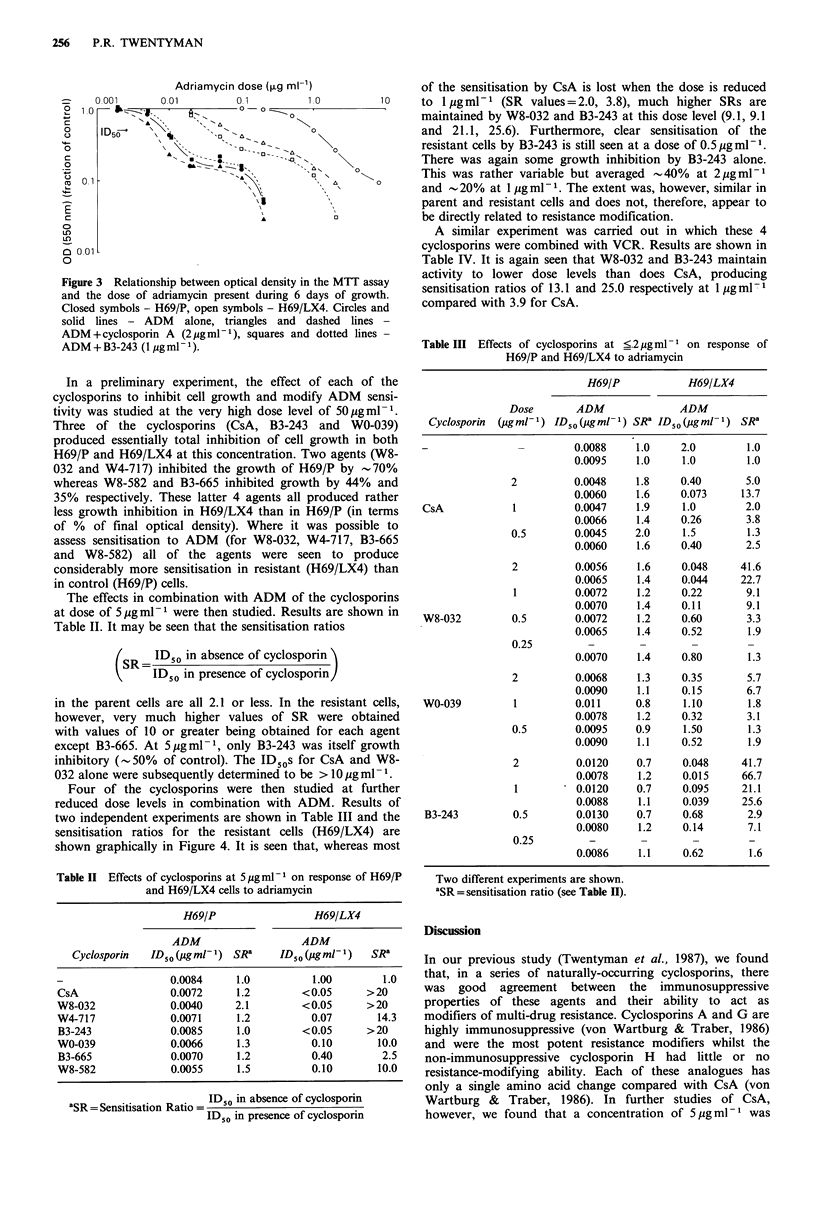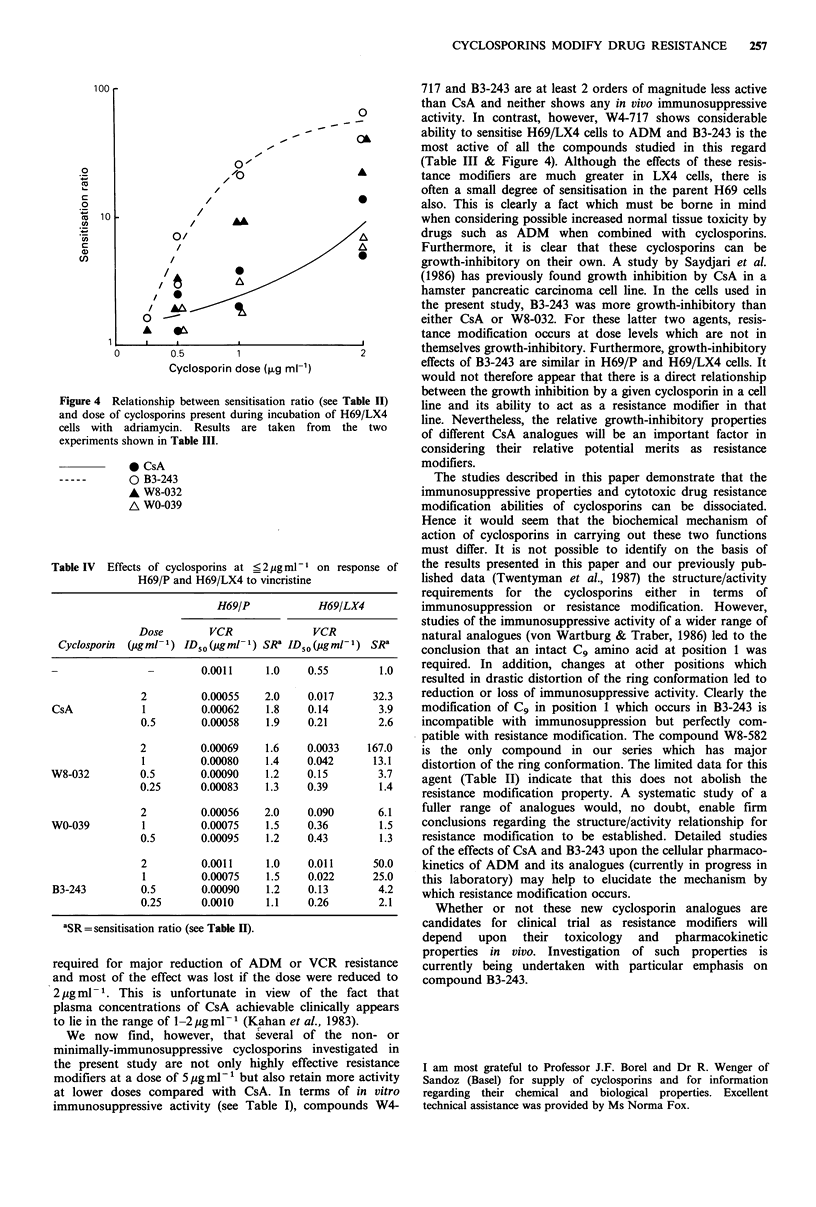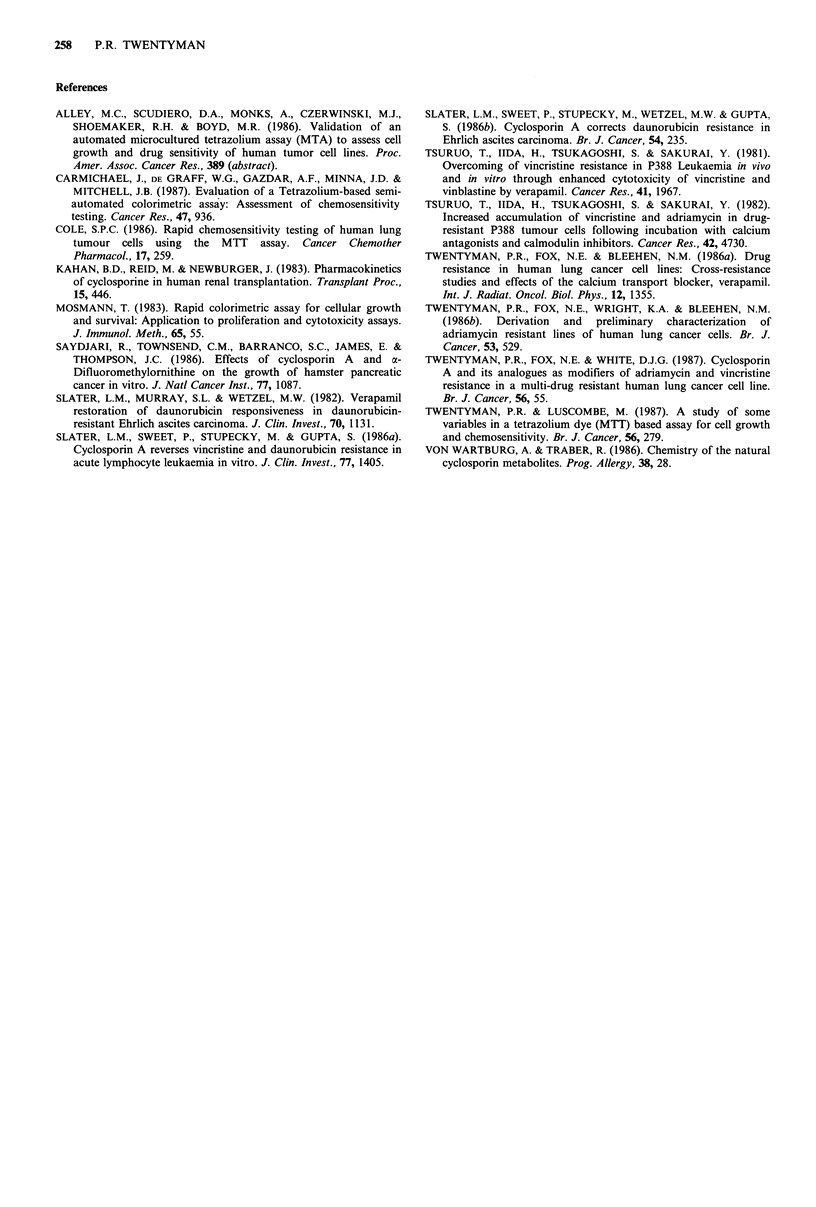# Modification of cytotoxic drug resistance by non-immuno-suppressive cyclosporins.

**DOI:** 10.1038/bjc.1988.55

**Published:** 1988-03

**Authors:** P. R. Twentyman

**Affiliations:** MRC Clinical Oncology and Radiotherapeutics Unit, Cambridge, UK.

## Abstract

We have examined the ability of a series of non- or minimally-immunosuppressive analogues of cyclosporin A to modify cytotoxic drug resistance in vitro. The series includes both cyclosporins derived from naturally-occurring compounds and synthetic cyclosporins. In contrast to our previous findings, we now report that several of these analogues are highly effective modifiers of resistance to adriamycin and vincristine in a multidrug resistant subline of the human small cell lung cancer cell line NCI-H69. Two of the analogues (W8-032 and B3-243) maintain considerable activity in the dose range 1-2 micrograms ml-1 whereas little activity remains for cyclosporin A when the dose is reduced to this level. B3-243, however, in contrast to cyclosporin A and W8-032, does itself show growth inhibitory effects in this dose range. Possible clinical trial of these cyclosporins as resistance modifiers will depend upon their in vivo toxicology and pharmacokinetic properties.


Br. J. Cancer (1988), 57, 254–258 © The Macmillan Press Ltd., 1988

### Modification of cytotoxic drug resistance by non-immuno-suppressive cyclosporins

P.R. Twentyman

*MRC Clinical Oncology and Radiotherapeutics Unit, Hills Road, Cambridge CB2 2QH, UK.*

**Summary** We have examined the ability of a series of non- or minimally-immunosuppressive analogues of cyclosporin A to modify cytotoxic drug resistance *in vitro*. The series includes both cyclosporins derived from naturally-occurring compounds and synthetic cyclosporins. In contrast to our previous findings, we now report that several of these analogues are highly effective modifiers of resistance to adriamycin and vincristine in a multidrug resistant subline of the human small cell lung cancer cell line NCI-H69. Two of the analogues (W8-032 and B3-243) maintain considerable activity in the dose range 1–2 $\mu g\,ml^{-1}$ whereas little activity remains for cyclosporin A when the dose is reduced to this level. B3-243, however, in contrast to cyclosporin A and W8-032, does itself show growth inhibitory effects in this dose range. Possible clinical trial of these cyclosporins as resistance modifiers will depend upon their *in vivo* toxicology and pharmacokinetic properties.

Pleiotropic drug resistance in model systems may be partially overcome by the use of additional chemical agents termed 'resistance modifiers'. The prototype agent is the calcium transport blocker, verapamil (Tsuruo *et al.*, 1981, 1982; Slater *et al.*, 1982; Twentyman *et al.*, 1986*a*). Recently, it has been shown by Slater *et al.* (1986*a,b*) that the immunosuppressive drug, cyclosporin A acts as a modifier of resistance to daunorubicin and vincristine (VCR) in Ehrlich ascites carcinoma *in vivo* and in human acute lymphatic leukaemia *in vitro*. Our studies of cyclosporin A in a highly multi-drug resistant variant of a human small cell lung cancer cell line (Twentyman *et al.*, 1987) confirmed that the agent could considerably reduce the degree of resistance to both adriamycin (ADM) and VCR. Three naturally-occurring analogues were also studied and a close correlation was seen between the immunosuppressive properties of the analogues and their ability to modify drug resistance. In an attempt to further clarify this relationship, we have now studied an additional series of cyclosporin analogues and the results of these studies are the subject of this paper.

### Materials and methods

### *Cells and culture conditions*

The cells used in this study were of the human small cell lung cancer line NCI-H69 and a highly multi-drug resistant subline (e.g. >100 fold resistance to ADM) derived in this laboratory (Twentyman *et al.*, 1986*a*). The parent cells are designated H69/P and the resistant subline H69/LX4. The cells are routinely grown in RPMI 1640 medium (Gibco Biocult) supplemented with 10% foetal calf serum (Seralab Ltd) together with penicillin and streptomycin. The cells grow as free-floating aggregates and the population doubling times of H69/P and H69/LX4 in the absence of drug are 39 (95% CL=33–47) and 54 (46–64) h respectively. Subline H69/LX4 is routinely maintained in the presence of 0.4 $\mu g\,ml^{-1}$ ADM but cells were washed free of drug and kept in drug-free medium for 2–3 days before use in experiments. Growing cultures of cells were mechanically disaggregated for experimental use by repeated pipetting. This resulted in a suspension consisting of single cells and small aggregates of up to 10 cells. From this mechanically-disaggregated suspension an aliquot was taken and formally disaggregated using trypsin and versene (Twentyman *et al.*, 1986*b*). A haemocytometer count was then carried out and the figure obtained was used as an estimate of the cell concentration in the mechanically-disaggregated suspension which was actually used for setting up experiments. This procedure was considered necessary as enzymatic reduction of H69 cultures to a single cell suspension results in a prolonged 'lag' in subsequent growth whereas mechanical disaggregation appears to be much less growth inhibitory.

### *Response assay*

To determine the drug response of H69/P and LX4 cells, we used a tetrazolium dye reduction assay (MTT) carried out on 96-well microtitre plates (Falcon Plastics). The assay is based on that originally described by Mosmann (1983) but using DMSO as the solvent (Alley *et al.*, 1986; Carmichael *et al.*, 1987). We have previously studied a number of variables involved in the optimal use of this assay (Twentyman & Luscombe, 1987). Evidence that the assay may be validly used to assess the response of human lung cancer cells to cytotoxic drugs has recently been published (Cole, 1986; Carmichael *et al.*, 1987).

The assay is based on the ability of viable cells to reduce a yellow-coloured soluble tetrazolium salt, 3–4, 5 dimethylthiazol-2, 5 diphenyl tetrazolium bromide (MTT) to an insoluble, purple-coloured formazan precipitate. After aspiration of most of the medium, the formazan may be dissolved in DMSO and the optical density measured at 550 nm using an ELISA plate reader (Twentyman & Luscombe, 1987). The assay represents a rapid and semi-automatic method of quantifying the number of viable cells/well following a period of growth in the presence or absence of various drugs or drug combinations.

For the experiments described in this paper, 96-well plates were set up with between $5\times10^3$ and $10^4$ (H69/P) or between $10^4$ and $2\times10^4$ (H69/LX4) cells per well in 200 $\mu l$ medium. After a period of ~2 h incubation (to allow equilibration of medium pH, etc), cyclosporins (or solvent controls) were added to each well in a volume of 10 $\mu l$. After 2 more hours of incubation, cytotoxic drugs (ADM or VCR) were added in 20 $\mu l$. To determine each dose-response curve we used one control well and seven 2-fold dilutions of cytotoxic drug. Within any single experiment, 3 or 4 replicates of each drug response point were carried out. Plates were then incubated for a period of 6 days in a gassing incubator (8% $CO_2$, 92% air) at 37°C. (This period was chosen as the time for control H69/P cells to have increased in number by 10 fold and be approaching the end of the exponential growth phase (Twentyman *et al.*, 1986*b*)).

At the end of the incubation period, 20 $\mu l$ of a 5 $mg\,ml^{-1}$ solution of MTT (Sigma) in PBS was added to each well and the plates returned to the incubator for a further 5 h. After this, the plates were centrifuged for 5 min at 400 $g$ in order to pack the floating cell aggregates to the bottom of the wells.

Received 18 September 1987; and in revised form, 4 January 1988.

The bulk of the medium was removed from each well using a Pasteur pipette connected to a vacuum line, leaving 10–20 µl medium per well. To each well was then added 200 µl DMSO and the plates were agitated on a plate-shaker for 5 min. Optical densities were then read at a wavelength of 540 nm on a 'Titertek Multiskan' ELISA plate reader.

*Drugs*

Cyclosporin A and 6 analogues were kindly supplied by Sandoz (Basel) Ltd. The structures of those compounds are shown in Figure 1 and their immunosuppressive properties in Table I. The cyclosporins were initially dissolved in absolute ethanol at a concentration of 5 mg ml$^{-1}$ and then diluted in medium before addition to wells. The final concentration of ethanol in the wells was 0.1% and control experiments showed that this neither affects cell growth or modifies the response to ADM or VCR in our system. In one preliminary experiment, cyclosporins were (mistakenly) added to wells in a final ethanol concentration of 1.0% but again we were able to show that this did not affect cell growth or ADM response. Upon dilution of the alcoholic solutions into medium, a fine precipitation of the cyclosporins was seen to occur. We were unwilling to use agents such as Tween 80 in order to prevent this because of the possibility that such membrane-active agents may themselves modify drug-resistance. It was considered that protein-binding of the cyclosporins would enable the drug to be presented to the cells in an acceptable state despite the apparent precipitation.

Cytotoxic drugs adriamycin (ADM, Pharmitalia) and vincristine (VCR, Eli Lilley) were initially dissolved in sterile water and solutions of 500 µg ml$^{-1}$ stored either at −20°C (ADM) or +4°C (VCR). Dilutions for use in experiments were prepared immediately before use.

**Figure 1** Amino acid sequence of cyclosporins. Redrawn from von Wartburg and Traber (1986), $C_9$ is a hitherto unknown amino acid = [2S, 3R, 4R, 6E]-3-hydroxy-4-methyl-2-methyl amino-6-octenoic acid; Abu = L-α-amino butyric acid; MeVal = N-methyl-L-valine; MeLeu = N-methyl-L-leucine.

**Table I** Immunosuppressive properties of cyclosporins[a]

| | | Immunosuppressive activity | |
|---|---|---|---|
| *Cyclosporin* | *Origin*[b] | *In vitro*[c] ($IC_{50}$ *µg ml*$^{-1}$) | *In vivo*[d] |
| Cyclosporin A | N | <0.04 | highly positive |
| W8-032 | S | 0.4–1.0 | negative |
| W4-717 | S | ≧5.0 | not tested |
| B3-243 | D | ≧5.0 | negative |
| W0-039 | S | 0.3–3.0 | negative |
| B3-665 | D | 1.0–2.0 | negative |
| W8-582 | S | 1.0–5.0 | not tested |

[a]Data are personal communications from J. Borel and R. Wenger of Sandoz (Basel) Ltd; [b]N = naturally occurring, D = derivated from a naturally occurring compound, S = synthetic analogue; [c]The following classical assays were used for determining the $IC_{50}$ *in vitro*: mitogen activation (e.g. Con A) of murine spleen cells, mixed lymphocyte reaction, cell-mediated lympholysis, Mishell-Dutton (PFC) assay, all with murine spleen cells, cytotoxicity to P-815 mastocytoma cells.; [d]The *in vivo* models used were: antibody formation as measured by the plaque-forming assay (PFC) in mice and rats, localised graft-*versus*-host reaction (popliteal lymph node weight) in rats, delayed-type hypersensitivity reaction to oxazolone or with T-cell clones in mice, adjuvant arthritis in rats, renal allograft in rats.

NB. Some compounds have not been tested in every assay.

### Results

We first of all studied the relationship in the MTT assay between the number of cells plated and the final optical density (OD) 6 days later. A typical set of results is shown in Figure 2. It may be seen that the relationship is approximately linear for both H69/P and H69/LX4 up to a cell number plated of $10^4$ (OD = 1.8) (H69/P) or $2 \times 10^4$ (OD = 1.4) (H69/LX4) with both lines passing through the origin. These data enabled us to select the optimal number of cells to plate in subsequent experiments. A typical set of dose-response data obtained using the MTT assay is shown in Figure 3. For each dose-response curve an $ID_{50}$ can be read as the drug dose required to reduce to final optical density to 50% of the control value. There is a very close agreement between several of the curves shown in Figure 3 and the corresponding curves which we have previously published using a 'total cell count assay' (Twentyman *et al.*, 1987) for fractional optical densities above 0.2–0.3. At higher drug doses, the MTT curves tend to show a plateau not seen when using total cell count as the endpoint. We believe that this plateau represents an artefact of the MTT assay and hence we have used the $ID_{50}$ to quantitate drug sensitivity using MTT whereas we previously used the $ID_{80}$.

**Figure 2** Relationship between optical density measured in the MTT assay at day 6 and the number of cells inoculated at day 0 into 96 well microtitre plates. Solid circles – H69/P, open circles – H69/LX4. Points are mean values of 4 replicate wells and error bars show the standard deviation. No error bars are shown when these are smaller than the dimensions of the symbol.

**Figure 3** Relationship between optical density in the MTT assay and the dose of adriamycin present during 6 days of growth. Closed symbols – H69/P, open symbols – H69/LX4. Circles and solid lines – ADM alone, triangles and dashed lines – ADM+cyclosporin A ($2\,\mu g\,ml^{-1}$), squares and dotted lines – ADM+B3-243 ($1\,\mu g\,ml^{-1}$).

In a preliminary experiment, the effect of each of the cyclosporins to inhibit cell growth and modify ADM sensitivity was studied at the very high dose level of $50\,\mu g\,ml^{-1}$. Three of the cyclosporins (CsA, B3-243 and W0-039) produced essentially total inhibition of cell growth in both H69/P and H69/LX4 at this concentration. Two agents (W8-032 and W4-717) inhibited the growth of H69/P by ~70% whereas W8-582 and B3-665 inhibited growth by 44% and 35% respectively. These latter 4 agents all produced rather less growth inhibition in H69/LX4 than in H69/P (in terms of % of final optical density). Where it was possible to assess sensitisation to ADM (for W8-032, W4-717, B3-665 and W8-582) all of the agents were seen to produce considerably more sensitisation in resistant (H69/LX4) than in control (H69/P) cells.

The effects in combination with ADM of the cyclosporins at dose of $5\,\mu g\,ml^{-1}$ were then studied. Results are shown in Table II. It may be seen that the sensitisation ratios

$$\left(SR = \frac{ID_{50} \text{ in absence of cyclosporin}}{ID_{50} \text{ in presence of cyclosporin}}\right)$$

in the parent cells are all 2.1 or less. In the resistant cells, however, very much higher values of SR were obtained with values of 10 or greater being obtained for each agent except B3-665. At $5\,\mu g\,ml^{-1}$, only B3-243 was itself growth inhibitory (~50% of control). The $ID_{50}$s for CsA and W8-032 alone were subsequently determined to be $>10\,\mu g\,ml^{-1}$.

Four of the cyclosporins were then studied at further reduced dose levels in combination with ADM. Results of two independent experiments are shown in Table III and the sensitisation ratios for the resistant cells (H69/LX4) are shown graphically in Figure 4. It is seen that, whereas most of the sensitisation by CsA is lost when the dose is reduced to $1\,\mu g\,ml^{-1}$ (SR values = 2.0, 3.8), much higher SRs are maintained by W8-032 and B3-243 at this dose level (9.1, 9.1 and 21.1, 25.6). Furthermore, clear sensitisation of the resistant cells by B3-243 is still seen at a dose of $0.5\,\mu g\,ml^{-1}$. There was again some growth inhibition by B3-243 alone. This was rather variable but averaged ~40% at $2\,\mu g\,ml^{-1}$ and ~20% at $1\,\mu g\,ml^{-1}$. The extent was, however, similar in parent and resistant cells and does not, therefore, appear to be directly related to resistance modification.

A similar experiment was carried out in which these 4 cyclosporins were combined with VCR. Results are shown in Table IV. It is again seen that W8-032 and B3-243 maintain activity to lower dose levels than does CsA, producing sensitisation ratios of 13.1 and 25.0 respectively at $1\,\mu g\,ml^{-1}$ compared with 3.9 for CsA.

**Table II** Effects of cyclosporins at $5\,\mu g\,ml^{-1}$ on response of H69/P and H69/LX4 cells to adriamycin

| | *H69/P* | | *H69/LX4* | |
|---|---|---|---|---|
| *Cyclosporin* | *ADM $ID_{50}$ ($\mu g\,ml^{-1}$)* | *SR*[a] | *ADM $ID_{50}$ ($\mu g\,ml^{-1}$)* | *SR*[a] |
| – | 0.0084 | 1.0 | 1.00 | 1.0 |
| CsA | 0.0072 | 1.2 | <0.05 | >20 |
| W8-032 | 0.0040 | 2.1 | <0.05 | >20 |
| W4-717 | 0.0071 | 1.2 | 0.07 | 14.3 |
| B3-243 | 0.0085 | 1.0 | <0.05 | >20 |
| W0-039 | 0.0066 | 1.3 | 0.10 | 10.0 |
| B3-665 | 0.0070 | 1.2 | 0.40 | 2.5 |
| W8-582 | 0.0055 | 1.5 | 0.10 | 10.0 |

[a]SR = Sensitisation Ratio = $\frac{ID_{50} \text{ in absence of cyclosporin}}{ID_{50} \text{ in presence of cyclosporin}}$

**Table III** Effects of cyclosporins at $\leq 2\,\mu g\,ml^{-1}$ on response of H69/P and H69/LX4 to adriamycin

| | | *H69/P* | | *H69/LX4* | |
|---|---|---|---|---|---|
| *Cyclosporin* | *Dose ($\mu g\,ml^{-1}$)* | *ADM $ID_{50}$ ($\mu g\,ml^{-1}$)* | *SR*[a] | *ADM $ID_{50}$ ($\mu g\,ml^{-1}$)* | *SR*[a] |
| – | – | 0.0088 | 1.0 | 2.0 | 1.0 |
| | | 0.0095 | 1.0 | 1.0 | 1.0 |
| CsA | 2 | 0.0048 | 1.8 | 0.40 | 5.0 |
| | | 0.0060 | 1.6 | 0.073 | 13.7 |
| | 1 | 0.0047 | 1.9 | 1.0 | 2.0 |
| | | 0.0066 | 1.4 | 0.26 | 3.8 |
| | 0.5 | 0.0045 | 2.0 | 1.5 | 1.3 |
| | | 0.0060 | 1.6 | 0.40 | 2.5 |
| W8-032 | 2 | 0.0056 | 1.6 | 0.048 | 41.6 |
| | | 0.0065 | 1.4 | 0.044 | 22.7 |
| | 1 | 0.0072 | 1.2 | 0.22 | 9.1 |
| | | 0.0070 | 1.4 | 0.11 | 9.1 |
| | 0.5 | 0.0072 | 1.2 | 0.60 | 3.3 |
| | | 0.0065 | 1.4 | 0.52 | 1.9 |
| | 0.25 | – | – | – | – |
| | | 0.0070 | 1.4 | 0.80 | 1.3 |
| W0-039 | 2 | 0.0068 | 1.3 | 0.35 | 5.7 |
| | | 0.0090 | 1.1 | 0.15 | 6.7 |
| | 1 | 0.011 | 0.8 | 1.10 | 1.8 |
| | | 0.0078 | 1.2 | 0.32 | 3.1 |
| | 0.5 | 0.0095 | 0.9 | 1.50 | 1.3 |
| | | 0.0090 | 1.1 | 0.52 | 1.9 |
| B3-243 | 2 | 0.0120 | 0.7 | 0.048 | 41.7 |
| | | 0.0078 | 1.2 | 0.015 | 66.7 |
| | 1 | 0.0120 | 0.7 | 0.095 | 21.1 |
| | | 0.0088 | 1.1 | 0.039 | 25.6 |
| | 0.5 | 0.0130 | 0.7 | 0.68 | 2.9 |
| | | 0.0080 | 1.2 | 0.14 | 7.1 |
| | 0.25 | – | – | – | – |
| | | 0.0086 | 1.1 | 0.62 | 1.6 |

Two different experiments are shown.
[a]SR = sensitisation ratio (see **Table II**).

### Discussion

In our previous study (Twentyman *et al.*, 1987), we found that, in a series of naturally-occurring cyclosporins, there was good agreement between the immunosuppressive properties of these agents and their ability to act as modifiers of multi-drug resistance. Cyclosporins A and G are highly immunosuppressive (von Wartburg & Traber, 1986) and were the most potent resistance modifiers whilst the non-immunosuppressive cyclosporin H had little or no resistance-modifying ability. Each of these analogues has only a single amino acid change compared with CsA (von Wartburg & Traber, 1986). In further studies of CsA, however, we found that a concentration of $5\,\mu g\,ml^{-1}$ was

**Figure 4** Relationship between sensitisation ratio (see **Table II**) and dose of cyclosporins present during incubation of H69/LX4 cells with adriamycin. Results are taken from the two experiments shown in **Table III**.

——— ● CsA
----- ○ B3-243
▲ W8-032
△ W0-039

**Table IV** Effects of cyclosporins at $\leq 2 \mu g ml^{-1}$ on response of H69/P and H69/LX4 to vincristine

| Cyclosporin | Dose ($\mu g ml^{-1}$) | *H69/P* VCR $ID_{50}$ ($\mu g ml^{-1}$) | *H69/P* SR[a] | *H69/LX4* VCR $ID_{50}$ ($\mu g ml^{-1}$) | *H69/LX4* SR[a] |
|---|---|---|---|---|---|
| – | – | 0.0011 | 1.0 | 0.55 | 1.0 |
| | 2 | 0.00055 | 2.0 | 0.017 | 32.3 |
| CsA | 1 | 0.00062 | 1.8 | 0.14 | 3.9 |
| | 0.5 | 0.00058 | 1.9 | 0.21 | 2.6 |
| | 2 | 0.00069 | 1.6 | 0.0033 | 167.0 |
| | 1 | 0.00080 | 1.4 | 0.042 | 13.1 |
| W8-032 | 0.5 | 0.00090 | 1.2 | 0.15 | 3.7 |
| | 0.25 | 0.00083 | 1.3 | 0.39 | 1.4 |
| | 2 | 0.00056 | 2.0 | 0.090 | 6.1 |
| W0-039 | 1 | 0.00075 | 1.5 | 0.36 | 1.5 |
| | 0.5 | 0.00095 | 1.2 | 0.43 | 1.3 |
| | 2 | 0.0011 | 1.0 | 0.011 | 50.0 |
| | 1 | 0.00075 | 1.5 | 0.022 | 25.0 |
| B3-243 | 0.5 | 0.00090 | 1.2 | 0.13 | 4.2 |
| | 0.25 | 0.0010 | 1.1 | 0.26 | 2.1 |

[a]SR = sensitisation ratio (see **Table II**).

required for major reduction of ADM or VCR resistance and most of the effect was lost if the dose were reduced to $2 \mu g ml^{-1}$. This is unfortunate in view of the fact that plasma concentrations of CsA achievable clinically appears to lie in the range of $1–2 \mu g ml^{-1}$ (Kahan *et al.*, 1983).

We now find, however, that several of the non- or minimally-immunosuppressive cyclosporins investigated in the present study are not only highly effective resistance modifiers at a dose of $5 \mu g ml^{-1}$ but also retain more activity at lower doses compared with CsA. In terms of *in vitro* immunosuppressive activity (see Table I), compounds W4-717 and B3-243 are at least 2 orders of magnitude less active than CsA and neither shows any *in vivo* immunosuppressive activity. In contrast, however, W4-717 shows considerable ability to sensitise H69/LX4 cells to ADM and B3-243 is the most active of all the compounds studied in this regard (Table III & Figure 4). Although the effects of these resistance modifiers are much greater in LX4 cells, there is often a small degree of sensitisation in the parent H69 cells also. This is clearly a fact which must be borne in mind when considering possible increased normal tissue toxicity by drugs such as ADM when combined with cyclosporins. Furthermore, it is clear that these cyclosporins can be growth-inhibitory on their own. A study by Saydjari *et al.* (1986) has previously found growth inhibition by CsA in a hamster pancreatic carcinoma cell line. In the cells used in the present study, B3-243 was more growth-inhibitory than either CsA or W8-032. For these latter two agents, resistance modification occurs at dose levels which are not in themselves growth-inhibitory. Furthermore, growth-inhibitory effects of B3-243 are similar in H69/P and H69/LX4 cells. It would not therefore appear that there is a direct relationship between the growth inhibition by a given cyclosporin in a cell line and its ability to act as a resistance modifier in that line. Nevertheless, the relative growth-inhibitory properties of different CsA analogues will be an important factor in considering their relative potential merits as resistance modifiers.

The studies described in this paper demonstrate that the immunosuppressive properties and cytotoxic drug resistance modification abilities of cyclosporins can be dissociated. Hence it would seem that the biochemical mechanism of action of cyclosporins in carrying out these two functions must differ. It is not possible to identify on the basis of the results presented in this paper and our previously published data (Twentyman *et al.*, 1987) the structure/activity requirements for the cyclosporins either in terms of immunosuppression or resistance modification. However, studies of the immunosuppressive activity of a wider range of natural analogues (von Wartburg & Traber, 1986) led to the conclusion that an intact $C_9$ amino acid at position 1 was required. In addition, changes at other positions which resulted in drastic distortion of the ring conformation led to reduction or loss of immunosuppressive activity. Clearly the modification of $C_9$ in position 1 which occurs in B3-243 is incompatible with immunosuppression but perfectly compatible with resistance modification. The compound W8-582 is the only compound in our series which has major distortion of the ring conformation. The limited data for this agent (Table II) indicate that this does not abolish the resistance modification property. A systematic study of a fuller range of analogues would, no doubt, enable firm conclusions regarding the structure/activity relationship for resistance modification to be established. Detailed studies of the effects of CsA and B3-243 upon the cellular pharmacokinetics of ADM and its analogues (currently in progress in this laboratory) may help to elucidate the mechanism by which resistance modification occurs.

Whether or not these new cyclosporin analogues are candidates for clinical trial as resistance modifiers will depend upon their toxicology and pharmacokinetic properties *in vivo*. Investigation of such properties is currently being undertaken with particular emphasis on compound B3-243.

I am most grateful to Professor J.F. Borel and Dr R. Wenger of Sandoz (Basel) for supply of cyclosporins and for information regarding their chemical and biological properties. Excellent technical assistance was provided by Ms Norma Fox.

### References

ALLEY, M.C., SCUDIERO, D.A., MONKS, A., CZERWINSKI, M.J., SHOEMAKER, R.H. & BOYD, M.R. (1986). Validation of an automated microcultured tetrazolium assay (MTA) to assess cell growth and drug sensitivity of human tumor cell lines. *Proc. Amer. Assoc. Cancer Res.*, **389** (*abstract*).

CARMICHAEL, J., DE GRAFF, W.G., GAZDAR, A.F., MINNA, J.D. & MITCHELL, J.B. (1987). Evaluation of a Tetrazolium-based semi-automated colorimetric assay: Assessment of chemosensitivity testing. *Cancer Res.*, **47**, 936.

COLE, S.P.C. (1986). Rapid chemosensitivity testing of human lung tumour cells using the MTT assay. *Cancer Chemother Pharmacol.*, **17**, 259.

KAHAN, B.D., REID, M. & NEWBURGER, J. (1983). Pharmacokinetics of cyclosporine in human renal transplantation. *Transplant Proc.*, **15**, 446.

MOSMANN, T. (1983). Rapid colorimetric assay for cellular growth and survival: Application to proliferation and cytotoxicity assays. *J. Immunol. Meth.*, **65**, 55.

SAYDJARI, R., TOWNSEND, C.M., BARRANCO, S.C., JAMES, E. & THOMPSON, J.C. (1986). Effects of cyclosporin A and α-Difluoromethylornithine on the growth of hamster pancreatic cancer in vitro. *J. Natl Cancer Inst.*, **77**, 1087.

SLATER, L.M., MURRAY, S.L. & WETZEL, M.W. (1982). Verapamil restoration of daunorubicin responsiveness in daunorubicin-resistant Ehrlich ascites carcinoma. *J. Clin. Invest.*, **70**, 1131.

SLATER, L.M., SWEET, P., STUPECKY, M. & GUPTA, S. (1986*a*). Cyclosporin A reverses vincristine and daunorubicin resistance in acute lymphocyte leukaemia in vitro. *J. Clin. Invest.*, **77**, 1405.

SLATER, L.M., SWEET, P., STUPECKY, M., WETZEL, M.W. & GUPTA, S. (1986*b*). Cyclosporin A corrects daunorubicin resistance in Ehrlich ascites carcinoma. *Br. J. Cancer*, **54**, 235.

TSURUO, T., IIDA, H., TSUKAGOSHI, S. & SAKURAI, Y. (1981). Overcoming of vincristine resistance in P388 Leukaemia *in vivo* and *in vitro* through enhanced cytotoxicity of vincristine and vinblastine by verapamil. *Cancer Res.*, **41**, 1967.

TSURUO, T., IIDA, H., TSUKAGOSHI, S. & SAKURAI, Y. (1982). Increased accumulation of vincristine and adriamycin in drug-resistant P388 tumour cells following incubation with calcium antagonists and calmodulin inhibitors. *Cancer Res.*, **42**, 4730.

TWENTYMAN, P.R., FOX, N.E. & BLEEHEN, N.M. (1986*a*). Drug resistance in human lung cancer cell lines: Cross-resistance studies and effects of the calcium transport blocker, verapamil. *Int. J. Radiat. Oncol. Biol. Phys.*, **12**, 1355.

TWENTYMAN, P.R., FOX, N.E., WRIGHT, K.A. & BLEEHEN, N.M. (1986*b*). Derivation and preliminary characterization of adriamycin resistant lines of human lung cancer cells. *Br. J. Cancer*, **53**, 529.

TWENTYMAN, P.R., FOX, N.E. & WHITE, D.J.G. (1987). Cyclosporin A and its analogues as modifiers of adriamycin and vincristine resistance in a multi-drug resistant human lung cancer cell line. *Br. J. Cancer*, **56**, 55.

TWENTYMAN, P.R. & LUSCOMBE, M. (1987). A study of some variables in a tetrazolium dye (MTT) based assay for cell growth and chemosensitivity. *Br. J. Cancer*, **56**, 279.

VON WARTBURG, A. & TRABER, R. (1986). Chemistry of the natural cyclosporin metabolites. *Prog. Allergy*, **38**, 28.